# Building integral projection models with nonindependent vital rates

**DOI:** 10.1002/ece3.8682

**Published:** 2022-03-21

**Authors:** Yik Leung Fung, Ken Newman, Ruth King, Perry de Valpine

**Affiliations:** ^1^ 34549 School of Mathematics University of Edinburgh Edinburgh UK; ^2^ 34549 Biomathematics and Statistics Scotland Edinburgh UK; ^3^ Department of Environmental Science, Policy and Management University of California Berkeley California USA

**Keywords:** copula models, correlated vital rates, generalized linear mixed models, population growth rate, reproduction investment, Soay sheep

## Abstract

Population dynamics are functions of several demographic processes including survival, reproduction, somatic growth, and maturation. The rates or probabilities for these processes can vary by time, by location, and by individual. These processes can co‐vary and interact to varying degrees, e.g., an animal can only reproduce when it is in a particular maturation state. Population dynamics models that treat the processes as independent may yield somewhat biased or imprecise parameter estimates, as well as predictions of population abundances or densities. However, commonly used integral projection models (IPMs) typically assume independence across these demographic processes. We examine several approaches for modelling between process dependence in IPMs and include cases where the processes co‐vary as a function of time (temporal variation), co‐vary within each individual (individual heterogeneity), and combinations of these (temporal variation and individual heterogeneity). We compare our methods to conventional IPMs, which treat vital rates independent, using simulations and a case study of Soay sheep (*Ovis aries*). In particular, our results indicate that correlation between vital rates can moderately affect variability of some population‐level statistics. Therefore, including such dependent structures is generally advisable when fitting IPMs to ascertain whether or not such between vital rate dependencies exist, which in turn can have subsequent impact on population management or life‐history evolution.

## Graphical abstract

The rate of demographic processes including survival, reproduction, somatic growth, and maturation can co‐vary and interact to varying degrees, e.g., an animal can only reproduce when it is in a particular maturation state. However, commonly used integral projection models (IPMs) typically assume independence across these demographic processes. We examine several approaches for modeling the between‐process dependence in IPMs and include cases where the processes co‐vary as a function of time (temporal variation), co‐vary within each individual (individual heterogeneity), and combinations of these (temporal variation and individual heterogeneity).

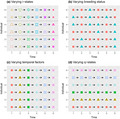

## 1 INTRODUCTION

Population models use estimated (or assumed) vital rates at the individual level to understand many aspects of a population's ecology and evolution: its long‐term abundance trajectory and age, size, or state distribution; its sensitivities and elasticities relevant for management; and its optimal life‐history strategy, among others. Variation in vital rates can have important affects on populations (Hamel et al., 2018; Vindenes & Langangen, 2015). This broad concept encompasses variation across individuals, across cohorts, and/or through time in ways described more below. In many models, potential variation in multiple vital rates is artificially assumed to be independent.

Looking beyond independent vital rates, ecologists have also long recognized the potential importance of nonindependent—i.e. correlated—vital rates on demography and life‐history evolution (Benton & Grant, 1999; Doak et al., 2005; Fieberg & Ellner, 2001). Correlations between growth, survival, reproduction, and/or other traits can change demographic conclusions (Coulson et al., 2005). For example, whereas independent temporal heterogeneity in vital rates has been generally predicted to decrease population growth rate, it can actually increase population growth rate when multiple vital rates are correlated (Doak et al., 2005). A completely different example is that persistent individual heterogeneity in vital rates can reveal different optimal life‐history strategies in different environmental conditions (Kentie et al., 2020).

Integral projection models (IPMs) are the framework for discrete‐time population dynamics with continuous individual‐state variables (e.g., mass, size) (Easterling et al., 2000). Compared to age‐ or stage‐structured matrix population models, which track abundance for each discrete state category, IPMs track abundance as a distribution (density) for continuous state values. This enables IPMs to more accurately represent populations in which continuous state variables are important predictors of individual dynamics such as growth, reproduction, and survival (Ellner et al., 2016; Merow et al., 2014; Rees et al., 2014). Thus, it may be important to incorporate both variation in vital rates and correlations among multiple vital rates into IPMs.

To what extent have correlated vital rates been incorporated into both estimation and analysis of IPMs? At a basic level, correlation in individual vital rates arising from stochastic life trajectories is almost inherent to a nontrivial IPM. For example, in a size‐structured IPM, correlation in growth and survival will arise when both depend on size and individual size trajectories vary due to stochastic growth. Temporal correlations among vital rates (e.g., a good year is good for each of growth, survival and reproduction) are captured naturally when year‐specific transition kernels are estimated or correlated random effects are estimated (Childs et al., 2004; Hindle et al., 2018; Metcalf et al., 2015). Correlations in individual heterogeneity among multiple traits have been considered for life‐history tradeoffs and eco‐evolutionary IPMs (Coulson et al., 2021; Kentie et al., 2020). However, there remains a need for systematic formulation and comparison of multiple kinds of correlated vital rates. This will allow for identification of gaps in statistical estimation and IPM analysis methods and comparison of impacts on demographic conclusions for the same data. Some IPM formulations have been sufficiently general to encompass these kinds of correlations from a mathematical perspective (Childs et al., 2016; Coulson et al., 2017), but case studies and estimation tools have not been as highly developed.

In this paper, the general concept of nonindependence among vital rates includes three quite different categories: (i) labile individual heterogeneity, (ii) temporal heterogeneity, and (iii) persistent individual heterogeneity. Labile individual heterogeneity refers to differences arising from phenotypic plasticity and the random events of a life course (Childs et al., 2016). This is also called dynamic condition (Forsythe et al., 2021) or transient heterogeneity (Brooks et al., 2017). For example, an individual who by luck experiences high‐growth conditions in early years may continue to be above average in size throughout its life. Labile heterogeneity can also arise from physiological tradeoffs such as costs of reproduction. For example, if an individual gives birth during the spring, its growth rate over subsequent months may be lower than if it had not given birth. In this example, the heterogeneity could be viewed as an individual‐level trade‐off between reproducing or growing more, although rigorously proving such causality cannot be done without a controlled experiment (Coulson, 2012; Knops et al., 2007). In statistical models, labile individual heterogeneity can be incorporated by making the transition (projection) kernels for multiple vital rates interdependent. Below, we consider both a standard regression framework and introduce a new copula approach for modelling such interdependence.

Temporal heterogeneity is driven by a shared covariate, which may be observed or unobserved (latent), that affects multiple traits (Compagnoni et al., 2016; Coulson et al., 2011; Hindle et al., 2018; Metcalf et al., 2015; Vindenes et al., 2014). For example, such a covariate could be annual (or breeding‐season) food supply that has a positive correlation with both survival probability and fecundity. Demographic data spanning multiple years would then show a positive correlation between population‐level survival and fecundity values. Note that a factor such as food supply could contribute to both temporal heterogeneity—to the extent individuals experience similar growth in a year due to the same conditions—and/or labile heterogeneity—to the extent individuals experience different growth due to heterogenous food conditions in the same year. We will present two different approaches for modelling correlated temporal heterogeneity, one being to explicitly include a shared and measured covariate that affects multiple vital rates and the other being to implicitly include shared but unmeasured covariates by including correlated temporal random effects.

Persistent individual heterogeneity in multiple traits refers to between‐individual differences that last their entire life (Brooks et al., 2017). This is also called fixed condition (Forsythe et al., 2021) or heterogeneity (Steiner et al., 2010). For example, one individual's average growth and fecundity rates could remain consistently higher than another individual's rates due to fixed heterogeneity. Persistent individual heterogeneity can be as simple as an univariate quality affecting a single trait (Ellner & Rees, 2006) or as complicated as a multivariate vector affecting the duration of the different life stages of an individual (de Valpine et al., 2014). Persistent individual heterogeneity is necessary to represent genetic variation in models of eco‐evolutionary dynamics (Childs et al., 2016; Vindenes & Langangen, 2015), but it can also represent only phenotypic variation potentially shaped by good site conditions at birth, for example. Processes such as energy acquisition allocation (van Noordwijk & de Jong, 1986) or reproductive strategy trade‐offs (Benton & Grant, 1999) could be considered as labile heterogeneity and/or persistent heterogeneity in different cases. In this paper, the statistical models of correlated persistent individual heterogeneity use correlated individual random effects (Brooks et al., 2017; Knape et al., 2011), although they can also use individual‐level covariates (Moyes et al., 2011). In summary, the three kinds of individual heterogeneity are biologically and statistically distinct, at least in principle.

Numerous IPM studies have incorporated one or more type of heterogeneity in vital rates, but few have incorporated nonindependent forms of heterogeneity (beyond the correlated vital rates arising from a basic IPM formulation). For example, Ellner and Rees (2006) incorporated persistent and labile individual heterogeneity without correlation, and Ellner and Rees (2006) incorporated temporal heterogeneity without correlation. As described by Vindenes and Langangen (2015), some studies include heterogeneity in estimation but then use only mean traits for analysis and prediction. Evolutionarily explicit IPMs have included both quantitative genetic traits and phenotypes as state variables, which together can be a kind of correlated persistent heterogeneity (Childs et al., 2016; Coulson et al., 2017, 2021; Rees & Ellner, 2019). Although these have mathematical similarity in IPM formulation, they are distinct in goals and statistical parameterisation methods compared with a nonevolutionary model with correlated individual traits. Kentie et al. (2020) considered correlated persistent heterogeneity among growth, survival, and reproduction, although they did not estimate these in a hierarchical statistical modeling framework as we do here. It is important to realize that each kind of correlated heterogeneity introduces different implementation challenges both for estimation and for IPM analysis involving multidimensional numerical integration, discussed more below.

Statistical estimation of different forms of nonindependent vital rates can draw on methods from other kinds of ecological analyses that, in some cases, have not typically been used for parameterization of IPMs. For labile individual heterogeneity, one current phenotypic value can be used to predict changes in another, which is basic to the formulation of IPMs. Such dependence can in principle include time lags, although these are not explored here. A potential limitation of the simple regression approach is that correlation among vital rates can be induced only be modifying the marginal distribution of the traits. We introduce the use of statistical copulas in this context as an alternative way to model labile correlations. For correlated temporal heterogeneity, one can include correlated temporal random effects or shared explanatory variables (Evans & Holsinger, 2012; Hindle et al., 2018; Metcalf et al., 2015). Alternatively, one can estimate different kernels for each of many years (Childs et al., 2004). Relevant to persistent individual heterogeneity, statistical models for individual demographic data routinely include random effects for individual heterogeneity, and multivariate random effects can be correlated (van Bonnet & Postma, 2016; de Pol & Verhulst, 2006). In the case of marked animals with imperfect detection or recapture, capture‐mark‐recapture methods can also incorporate correlated individual random effects (Cam et al., 2013; Gimenez et al., 2018).

In this paper, we systematically present statistical methods to estimate different kinds of correlations in vital rates and incorporate those correlations into IPMs. We give methods for modelling correlations in vital rate arising in each of the three categories of heterogeneity, including a new copula method for individual heterogeneity. We show how the methods can be used in a hierarchical statistical framework and discuss some of the computational and implementation challenges involved. In a case study with Soay sheep data, we illustrate that the same data can imply different demographic conclusions when different kinds of correlated vital rates are considered. In addition, even when including correlations does not change point results such as population growth rate or elasticities, it can change the width of uncertainty (credible or confidence interval) propagated from uncertainties in parameter estimates.

The structure of this paper is the following. We begin with a general description of IPMs (Section 2.1) and consider IPMs with independent vital rates (Section 2.2). We next discuss the area of primary focus: IPMs with heterogeneous and nonindependent vital rates (Section 2.3). We note here that while dependency and correlation are not exactly equivalent, we will use the terms interchangeably because of common practice. This is followed by a description of simulation studies and a case study using data from a population of Soay sheep (*Ovis aries*) in Scotland (Sections 2.5 and 2.6). The results of these studies (Section 3) focus on differences arising from the nonindependent vital rate models on (i) the log population growth rate and (ii) population growth rate elasticities. We conclude with a discussion of the implications of the proposed methods (Section 4).

## 2 METHODS

### 2.1 General integral projection models

We begin with a description of a family of IPMs that permit the incorporation of temporal, persistent and/or labile individual heterogeneity, using the notation from Childs et al. (2016). Let x denotes the individual state variables, hereafter called “i‐states.” The i‐states comprise labile traits that vary over the life cycle in response to the environment such as body mass, length or breeding status (Coulson, 2012; Merow et al., 2014; Rees et al., 2014). In addition, individuals are further characterised by “q‐states,” denoted by z. The q‐states comprise unmeasured, nonlabile characteristics that are fixed during the lifetime of the individual. In this article, we assume that (i) individuals can be uniquely characterized by (x,z), which essentially assumes that individuals with the same (x,z) are interchangeable, (ii) all vital rate models depend on x, and (iii) selected vital rate models depend on z. The values of (x,z) at one discrete time step later are denoted as (*x*′, *z*′).

The state of the population is described by the abundance density, denoted n(x,z,t). The abundance density is defined such that the number of individuals at time t with states in a small interval (x,z) to (x+Δx,z+Δz) is approximately n(x,z,t)ΔxΔz. The total abundance at t can then be expressed as $N_{t}$, such that

(1) $$N_{t}=\int \int n\left(x,z,t\right)dxdz.$$

The projection of the abundance density over time is described by the following equation,

(2) $$n\left(x^{\prime },z^{\prime },t+1\right)=\iint n(x,z,t)k\left(x^{\prime },z^{\prime }|x,z,d_{t}\right)dxdz,$$

where $k(x^{\prime },z^{\prime }|x,z,d_{t})$ is the time‐varying projection (transition) kernel, i.e. the density of individuals evolving from (x,z) to (*x*′, *z*′) (Ellner & Rees, 2007). The term $d_{t}$ denotes measured and/or unmeasured time‐specific environmental conditions that account for temporal variation. The functional form of the projection kernel depends on the parameterization of vital rate models and the life cycle of the study species. In this article, the formulation of the projection kernel is motivated by the life cycle of Soay sheep (Clutton‐Brock & Pemberton, 2004; Coulson, 2012) such that,

(3) $$k\left(x^{\prime },z^{\prime }|x,z,d_{t}\right)=s\left(x,z,d_{t}\right)\left[b\left(x,z,d_{t}\right)h\left(x^{\prime },z^{\prime }|x,z,d_{t}\right)+g\left(x^{\prime },z^{\prime }|x,z,d_{t}\right)\right],$$

where s(·) denotes survival probability; b(·) is the number of offspring of survived individuals; h(·) is the density of offspring with (*x*′, *z*′) from a reproducing individual with (x,z); and g(·) is the density of individuals growing from (x,z) to (*x*′, *z*′). The IPM kernel is a large‐population approximation, so these rates are expected values. Most births of Soay sheep are singletons, and for simplicity, we ignore twinning (Coulson, 2012).

In the following sections, we discuss different ways to construct vital rate models when rates are independent or dependent, given the i‐states, x. Motivated by reproduction cost (Gittleman & Thompson, 1988; Tavecchia et al., 2005), we restrict attention to the dependence between growth and reproduction.

### 2.2 Independent vital rate models

Before describing different formulations of vital rate models, we introduce some additional notation. To begin, we assume that there is only one element in the labile traits, x, and that is the natural logarithm of body mass. For individual j at time t, let $m_{j,t}$ denotes the log body mass (given survival); $a_{j,t}$ the alive (1) vs dead (0) state; $r_{j,t}$ the reproductive (1) vs nonreproductive (0) state (given survival); and $c_{j,t}$ the offspring log body mass (given reproduction). The discrete times are t=1,⋯,T.

In terms of parameters, fixed‐effect parameters are referenced as β with subscripts defining the vital rate and the variable they influence, respectively. For instance, $\beta_{g,0}$ is the intercept for the growth model and $\beta_{s,m}$ is the slope for the survival model corresponding to the variable m. Also, residual (nonrandom effect) variances are denoted by $\sigma^{2}$ with the subscript defining the vital rate. In addition to fixed effects, we consider random effects on year and individual for temporal and persistent individual heterogeneity, respectively. These random effects are placed on the growth and reproduction models to capture the potential dependence of interest. The unobserved temporal or individual random effects are denoted by u and v, respectively. For example, $u_{b,t}$ is the reproduction random year effect in year t, while $v_{g,j}$ is the growth random individual effect on individual j. Random effect variances are denoted by $\nu^{2}$ and $\theta^{2}$, and correlation parameters by ρ and ψ, respectively.

Assuming independence between vital rates, parameters for each vital rate model can be estimated separately. For that case, we summarize three of the most commonly used approaches to formulate vital rate models.

#### 2.2.1 Vanilla Model ( I1)

We initially define the “vanilla model,” denoted as model I1, as the widely used approach where the vital rates depend only on the labile phenotype, x, corresponding to the log body mass (m) in our Soay sheep example (Easterling et al., 2000; Ellner & Rees, 2006). In particular, parameters are estimated given the individual‐level demographic data such that,

(4) $$\begin{array}{cc} a_{j,t+1}|m_{j,t} & :\mathrm{Bernoulli}({\mathrm{logit}}^{‐1}\left(\beta_{s,0}+\beta_{s,m}m_{j,t}\right))\\ r_{j,t+1}|m_{j,t} & :\mathrm{Bernoulli}({\mathrm{logit}}^{‐1}\left(\beta_{b,0}+\beta_{b,m}m_{j,t}\right))\\ m_{j,t+1}|m_{j,t} & :N(\beta_{g,0}+\beta_{g,m}m_{j,t},\sigma_{g}^{2})\\ c_{j,t+1}|m_{j,t} & :N(\beta_{h,0}+\beta_{h,m}m_{j,t},\sigma_{h}^{2}), \end{array}$$

where ${\mathrm{logit}}^{‐1}\left(a\right)=1/\left(1+e^{‐a}\right)$ is the inverse of the logistic transformation. To apply the vanilla model to the projection kernel in Equation (3), we rearrange the vital rate models such that,

(5) $$\begin{array}{cc} s\left(m\right) & ={\text{logit}}^{‐1}\left(\beta_{s,0}+\beta_{s,m}m\right)\\ b\left(m\right) & ={\text{logit}}^{‐1}\left(\beta_{b,0}+\beta_{b,m}m\right)\\ g(m^{\prime }|m) & \equiv \phi \left(m^{\prime };\beta_{g,0}+\beta_{g,m}m,\sigma_{g}^{2}\right)\\ h(m^{\prime }|m) & \equiv \phi \left(m^{\prime };\beta_{h,0}+\beta_{h,m}m,\sigma_{h}^{2}\right), \end{array}$$

where $\phi (a;\mu ,\sigma^{2})$ denotes the density function of $N(\mu ,\sigma^{2})$ evaluated at a. Here, x=m, and there is no z or $d_{t}$. The equation for h(·) represents an inheritance or the “parent‐offspring phenotypic similarity” function (Coulson et al., 2021), with offspring size depending on parent size. For the following models, we assume the same vital rate models as described above if they are not mentioned in the model description.

#### 2.2.2 Temporal Heterogeneity ( I2)

Models with temporal heterogeneity connect vital rates with time‐varying factors, such as resource availability, natural enemies, and abiotic conditions. We consider a hierarchical model with independent random effects (Bolker et al., 2009; McCulloch & Searle, 2001) such that,

(6) $$\begin{array}{cc} r_{j,t+1}|m_{j,t},u_{b,t} & :\mathrm{Bernoulli}({\mathrm{logit}}^{‐1}\left(\beta_{b,0}+\beta_{b,m}m_{j,t}+u_{b,t}\right))\\ m_{j,t+1}|m_{j,t},u_{g,t} & :N(\beta_{g,0}+\beta_{g,m}m_{j,t}+u_{g,t},\sigma_{g}^{2})\\ u_{b,t} & :N(0,\nu_{b}^{2})\\ u_{g,t} & :N(0,\nu_{g}^{2}), \end{array}$$

where the random effects $u_{b,t}$ and $u_{g,t}$ are independent to avoid inducing dependence between different vital rate models.

Similar to Equation (5), the vital rate models are rearranged such that,

(7) $$\begin{array}{cc} b\left(m,u_{b,t}\right) & ={\text{logit}}^{‐1}\left(\beta_{b,0}+\beta_{b,m}m+u_{b,t}\right)\\ g(m^{\prime }|m,u_{g,t}) & \equiv \phi \left(m^{\prime };\beta_{g,0}+\beta_{g,m}m+u_{g,t},\sigma_{g}^{2}\right). \end{array}$$

Here, x=m, $d_{t}=\left(u_{b,t},u_{g,t}\right)$, and there is no z.

#### 2.2.3 Persistent Individual Heterogeneity ( I3)

The persistent individual heterogeneity model, denoted I3, differs from the temporal heterogeneity model (I2) by including random effects for each individual instead of each time step. The individual random effects represent phenotypic variability that persists through each individual's life. In particular, we specify,

(8) $$\begin{array}{cc} r_{j,t+1}|m_{j,t},v_{b,j} & :\mathrm{Bernoulli}({\mathrm{logit}}^{‐1}\left(\beta_{b,0}+\beta_{b,m}m_{j,t}+v_{b,j}\right))\\ m_{j,t+1}|m_{j,t},v_{g,j} & :N(\beta_{g,0}+\beta_{g,m}m_{j,t}+v_{g,j},\sigma_{g}^{2})\\ v_{b,j} & :N(0,\theta_{b}^{2})\\ v_{g,j} & :N(0,\theta_{g}^{2}), \end{array}$$

where the random effect distributions are independent to avoid inducing dependence. In this case, the vital rate models are re‐arranged as,

(9) $$\begin{array}{cc} b\left(m,v_{b}\right) & ={\text{logit}}^{‐1}\left(\beta_{b,0}+\beta_{b,m}m+v_{b}\right)\\ g(m^{\prime },v_{g^{\prime }}|m,v_{g}) & \equiv \phi \left(m^{\prime };\beta_{g,0}+\beta_{g,m}m+v_{g},\sigma_{g}^{2}\right)I\left(v_{g^{\prime }}=v_{g}\right)\\ h(m^{\prime },v_{b}^{o},v_{g}^{o}|m) & \equiv \phi \left(m^{\prime };\beta_{h,0}+\beta_{h,m}m,\sigma_{h}^{2}\right)\phi \left(v_{b}^{o};0,\theta_{b}^{2}\right)\phi \left(v_{g}^{o};0,\theta_{g}^{2}\right), \end{array}$$

where $v_{b}^{o}$ and $v_{g}^{o}$ denote the random individual effects for the offspring. Here, x=m, $z=(v_{b},v_{g})$, and there is no $d_{t}$. We assume offspring size depends on parent size while offspring random effects are independent of parent random effects.

### 2.3 Nonindependent vital rate models

We now discuss different ways to induce the dependence structure between vital rate models. Corresponding to the three types of heterogeneity are three categories of models, with a category representing labile individual heterogeneity having two models (D1a and D1b), the temporal heterogeneity category having two models (D2a and D2b), and the persistent individual heterogeneity category having one model (D3).

#### 2.3.1 Labile Individual Heterogeneity (D1a and D1b)

Models in this category extend the vanilla model I1 to create dependence between reproduction and growth. We construct two types of dependent vital rate models: (i) the reproduction conditional model and (ii) the copula model. The former model treats breeding status as a covariate within the growth model, while the latter model utilizes the copula structure to jointly model growth and reproduction. The latter necessitates estimating multiple kernel functions together, while the former does not.

##### D1a. Reproduction conditional model

This approach models the growth rate of an individual as a function of the breeding status. In particular, the binary variable, $r_{t+1,j}$, is a covariate in the growth model such that,

(10) $$m_{j,t+1}|m_{j,t},r_{j,t+1}:N\left(\beta_{g,0}+\beta_{g,m}m_{j,t}+\beta_{g|r}r_{j,t+1},\sigma_{g}^{2}\right).$$

Integrating out $r_{j,t+1}$ to obtain the marginal growth model for the projection kernel, we note that,

(11) $$\begin{array}{cc} g(m^{\prime }|m) & =b\left(m\right)\phi \left(m^{\prime };\beta_{g,0}+\beta_{g,m}m+\beta_{g|r},\sigma_{g}^{2}\right)+\left[1‐b\left(m\right)\right]\phi \left(m^{\prime };\beta_{g,0}+\beta_{g,m}m,\sigma_{g}^{2}\right), \end{array}$$

where the marginal growth distribution is now a mixture of two Gaussian distributions and hence potentially bimodal. Here, x=(m,r), and there is no z and $d_{t}$.

This model induces a dependency between growth and reproduction that is reflected in the covariance, $\text{cov}\left(m^{\prime },r^{\prime }\right)=\beta_{g|r}\text{var}\left(r^{\prime }\right)=\beta_{g|r}b\left(m\right)\left[1‐b\left(m\right)\right]$. This covariance is maximized when b(m)=0.5 and minimized as b(m) approaches 0 or 1.

##### D1b. Copula model

Copula methods are a popular approach to construct a joint distribution for correlated random variables given assumed marginal distributions [see, e.g., Chapter 6 of Song, 2007). These models extend univariate linear models to general multivariate models with vector responses and provide a flexible approach to the regression analysis of correlated discrete, continuous, or mixed responses (Anderson et al., 2019; de Valpine et al., 2014).

The copula method relies on Sklar's theorem (Sklar, 1959) which states that any multivariate distribution can be constructed by combining the marginal distributions with a suitable copula function describing the association between the variables. Mathematically, given the marginal cumulative distribution function (CDF) $F_{1}\left(\cdot \right),\cdots ,F_{n}\left(\cdot \right)$ of variables $Y_{1},\cdots ,Y_{n}$, and a copula function C, the joint CDF can be expressed as,

(12) $$F_{1,\cdots ,n}\left(y_{1},\cdots ,y_{n}\right)=P\left(Y_{1}\leq y_{1},\cdots ,Y_{n}\leq y_{n}\right)=C\left(P\left(Y_{1}\leq y_{1}\right),\cdots ,P\left(Y_{n}\leq y_{n}\right)\right),$$

where $F_{i}\left(y\right)=P\left(Y_{i}\leq y\right)$, i=1⋯n.

There are a variety of copula functions available that permit different behaviours of multidimensional distributions and typically lead to different dependence structures. However, the marginal distributions of the random variables remain the same irrespective of the choice of copula function. We use the Gaussian copula function to handle the dependence structure for simplicity (Nelsen, 2006; Song et al., 2009). The Gaussian copula function is defined such that,

(13) $$\begin{array}{cc} F_{1,\cdots ,n}\left(y_{1},\cdots ,y_{n}\right) & =\Phi_{D}\left\{\Phi^{‐1}\left[F_{1}\left(y_{1}\right)\right],\cdots ,\Phi^{‐1}\left[F_{n}\left(y_{n}\right)\right]\right\}\\ f_{1,\cdots ,n}\left(y_{1},\cdots ,y_{n}\right) & =\phi_{D}\left\{\Phi^{‐1}\left[F_{1}\left(y_{1}\right)\right],\cdots ,\Phi^{‐1}\left[F_{n}\left(y_{n}\right)\right]\right\}\prod_{i=1}^{n}\frac{f_{i}\left(y_{i}\right)}{\phi (\Phi^{‐1}\left(F_{i}\left(y_{i}\right)\right))}, \end{array}$$

where $\Phi^{‐1}\left(\cdot \right)$ denotes the inverse CDF of a standard Gaussian distribution; $\Phi_{D}\left(\cdot \right)$ and $\phi_{D}\left(\cdot \right)$ are the CDF and density, respectively, of a n‐dimensional Gaussian distribution with a zero vector as mean and covariance matrix D. The diagonal elements of D are all scaled to unity without the loss of generality.

As an example, we briefly describe the copula model used in the Soay sheep case study for correlated growth and reproduction, involving the combination of a continuous and discrete random variable. In particular, we use the Gaussian copula function with a normally distributed random variable for growth, $Y_{1}$, and a Bernoulli‐distributed random variable for reproduction, denoted $Y_{2}$. Note that the density function and CDF of $Y_{1}$ are expressed as,

(14) $$\begin{array}{cc} f_{1}\left(y_{1}\right) & =\phi (y_{1};\mu ,\sigma^{2})\\ F_{1}\left(y_{1}\right) & =\Phi \left(\frac{y_{1}‐\mu }{\sigma }\right), \end{array}$$

where μ is the expected value of $Y_{1}$, and $\sigma^{2}$ is the variance of $Y_{1}$. For the reproduction (Bernoulli) variable, as the raw scale is discrete, we introduce an auxiliary variable X, which is distributed as an uniform distribution (i.e., X:U[0,1]) and define the new random variable $Y_{3}=Y_{2}+X$. The probability mass function for $Y_{2}$, the probability density function for $Y_{3}$, and the CDFs for both are then expressed as,

(15) $$\begin{array}{c} f_{2}\left(y_{2}\right)=\left\{\begin{array}{cc} q & \;\text{if}\;y_{2}=0\\ 1‐q & \;\text{if}\;y_{2}=1\\ 0 & \;\text{otherwise}\; \end{array}\right.\;f_{3}\left(y_{3}\right)=\left\{\begin{array}{cc} q & \;\text{if}\;0\leq y_{3}<1\\ 1‐q & \;\text{if}\;1\leq y_{3}\leq 2\\ 0 & \;\text{otherwise}\; \end{array}\right.\\ \Rightarrow \\ F_{2}\left(y_{2}\right)=\left\{\begin{array}{cc} 0 & \;\text{if}\;y_{2}<0\\ q & \;\text{if}\;0\leq y_{2}<1\\ 1 & \;\text{if}\;y_{2}\geq 1 \end{array}\right.\;F_{3}\left(y_{3}\right)=\left\{\begin{array}{cc} 0 & \;\text{if}\;y_{3}<0\\ qy_{3} & \;\text{if}\;0\leq y_{3}<1\\ q+\left(1‐q\right)\left(y_{3}‐1\right) & \;\text{if}\;1\leq y_{3}\leq 2\\ 1 & \;\text{if}\;y_{3}\geq 2 \end{array}\right. \end{array}$$

where $q=Pr\left(Y_{2}=0\right)$. Combining Equations (13) and (15), we derive the joint density of $\left(Y_{1},Y_{3}\right)$ such that,

(16) $$\begin{array}{c} f\left(y_{1},y_{3}\right)\equiv \phi_{D}\left\{\frac{y_{1}‐\mu }{\sigma },\Phi^{‐1}\left[F_{3}\left(y_{3}\right)\right]\right\}\frac{1}{\sigma }\frac{f_{3}\left(y_{3}\right)}{\phi \left(\Phi^{‐1}\left(F_{3}\left(y_{3}\right)\right)\right)}. \end{array}$$

We can then substitute the growth and reproduction model for $Y_{1}$ and $Y_{2}$ to obtain their corresponding joint density for parameter estimation. The notation becomes $x=\left(m,r\right)$, and there is no z and $d_{t}$.

Despite the appealing features of copula models, IPMs with copula models give the same projection kernel as the vanilla model, which leads to the identical projection of the population dynamics. This is true because (i) correlations in the copula model do not modify the marginal distributions and (ii) the involved vital rate models (reproduction and growth) are an additive structure. Further details are presented in Appendix S1. Demographically, population change is the same whether individuals who grow less are the ones who reproduced more or not. However, as discussed more below, the copula remains interesting because it may give different answers for life‐history questions involving trade‐offs, or estimated parameters may be different, or it may give different kernels when used with time lags or other extensions.

#### 2.3.2 Temporal heterogeneity ( D2a and D2b)

These models induce dependence on vital rates by the time‐varying factors, extending the independent temporal heteroegeneity model, I2. In particular, when the conditions of a given year are “good” for both growth and reproduction, temporal heterogeneity will create positive temporal correlation among these vital rates, which may generally be the case (Hindle et al., 2018). We consider two models: (i) the shared drivers model and (ii) the correlated random year effect model. The former model accounts for the temporal effect explicitly with additional covariate(s), while the latter model utilizes random year effects to implicitly model the impacts of unknown temporal factors.

##### D2a. Shared drivers model

This approach includes observed time‐varying covariates in the regression functions for vital rate models (van Benthem et al., 2017; Dalgleish et al., 2011; Simmonds & Coulson, 2015). Common choices include environmental indices, e.g., North Atlantic Oscillation, precipitation, temperature, etc. To quantify the additional influence of the drivers on the vital rates, let $q_{t}$ denotes the vector of covariates with an associated vector of regression coefficients $\beta_{\cdot ,q}$, namely

(17) $$\begin{array}{cc} r_{j,t+1}|m_{j,t},q_{t} & :\text{Bernoulli}\left({\text{logit}}^{‐1}\left(\beta_{b,0}+\beta_{b,m}m_{j,t}+\beta_{b,q}q_{t}\right)\right)\\ m_{j,t+1}|m_{j,t},q_{t} & :N\left(\beta_{g,0}+\beta_{g,m}m_{j,t}+\beta_{g,q}q_{t},\sigma_{g}^{2}\right). \end{array}$$

The vital rate models are re‐arranged for the projection kernel such that,

(18) $$\begin{array}{cc} b\left(m,q_{t}\right) & ={\text{logit}}^{‐1}\left(\beta_{b,0}+\beta_{b,m}m+\beta_{b,q}q_{t}\right)\\ g(m^{\prime }|m,q_{t}) & \equiv \phi \left(m^{\prime };\beta_{g,0}+\beta_{g,m}m+\beta_{g,q}q_{t},\sigma_{g}^{2}\right). \end{array}$$

Here, x=m, $d_{t}=q_{t}$, and there is no z.

##### D2b. Correlated random year effect model

The second model extends the independent temporal random effects model (model I2) Generalizing these hierarchical models by allowing for dependencies in the random effect distributions induces dependencies between vital rates (Hindle et al., 2018; Metcalf et al., 2015) such that,

(19) $$\begin{array}{cc} r_{j,t+1}|m_{j,t},u_{b,t} & :\text{Bernoulli}\left({\text{logit}}^{‐1}\left(\beta_{b,0}+\beta_{b,m}m_{j,t}+u_{b,t}\right)\right)\\ m_{j,t+1}|m_{j,t},u_{g,t} & :N\left(\beta_{g,0}+\beta_{g,m}m_{j,t}+u_{g,t},\sigma_{g}^{2}\right)\\ \left(\begin{array}{c} u_{b,t}\\ u_{g,t} \end{array}\right) & :N\left[\begin{array}{c} \left(\begin{array}{c} 0\\ 0 \end{array}\right),\left(\begin{array}{cc} \nu_{b}^{2} & \rho \nu_{b}\nu_{g}\\ \rho \nu_{b}\nu_{g} & \nu_{g}^{2} \end{array}\right) \end{array}\right]. \end{array}.$$

The vital rate models are re‐arranged for the projection kernel such that,

(20) $$\begin{array}{cc} b\left(m,u_{b,t}\right) & ={\text{logit}}^{‐1}\left(\beta_{b,0}+\beta_{b,m}m+u_{b,t}\right)\\ g(m^{\prime }|m,u_{g,t}) & \equiv \phi \left(m^{\prime };\beta_{g,0}+\beta_{g,m}m+u_{g,t},\sigma_{g}^{2}\right). \end{array}$$

Here, x=m, $d_{t}=\left(u_{b,t},u_{g,t}\right)$, and there is no z.

#### 2.3.3 Persistent individual heterogeneity ( D3)

Similar to the temporal heterogeneity, the model in this category extends model I3 to induce dependence between vital rates for the persistent individual heterogeneity case.

##### D3. Correlated random individual effect model

We consider a hierarchical model with dependent random effects distribution, similar to model D2b. In particular, we specify,

(21) $$\begin{array}{cc} r_{j,t+1}|m_{j,t},v_{b,j} & :\text{Bernoulli}\left({\text{logit}}^{‐1}\left(\beta_{b,0}+\beta_{b,m}m_{j,t}+v_{b,j}\right)\right)\\ m_{j,t+1}|m_{j,t},v_{g,j} & :N\left(\beta_{g,0}+\beta_{g,m}m_{j,t}+v_{g,j},\sigma_{g}^{2}\right)\\ \left(\begin{array}{c} v_{b,j}\\ v_{g,j} \end{array}\right) & :N\left[\begin{array}{c} \left(\begin{array}{c} 0\\ 0 \end{array}\right),\left(\begin{array}{cc} \theta_{b}^{2} & \psi \theta_{b}\theta_{g}\\ \psi \theta_{b}\theta_{g} & \theta_{g}^{2} \end{array}\right) \end{array}\right]. \end{array}$$

The vital rate models are re‐arranged for the projection kernel such that,

(22) $$\begin{array}{cc} b\left(m,v_{b}\right) & ={\text{logit}}^{‐1}\left(\beta_{b,0}+\beta_{b,m}m+v_{b}\right)\\ g(m^{\prime },v_{g^{\prime }}|m,v_{g}) & \equiv \phi \left(m^{\prime };\beta_{g,0}+\beta_{g,m}m+v_{g},\sigma_{g}^{2}\right)I\left(v_{g^{\prime }}=v_{g}\right)\\ h(m^{\prime },v_{b}^{o},v_{g}^{o}|m) & \equiv \phi \left(m^{\prime };\beta_{h,0}+\beta_{h,m}m,\sigma_{h}^{2}\right)\phi_{\mathrm{ind}}\left(v_{b}^{o},v_{g}^{o}\right), \end{array}$$

where $\phi_{\mathrm{ind}}\left(\cdot \right)$ is the density function of the random individual effects distribution and specified in the last part of Equation (21). Here, x=m, $z=\left(v_{b},v_{g}\right)$, and there is no $d_{t}$.

#### 2.3.4 Comparison of the models

**FIGURE 1:**
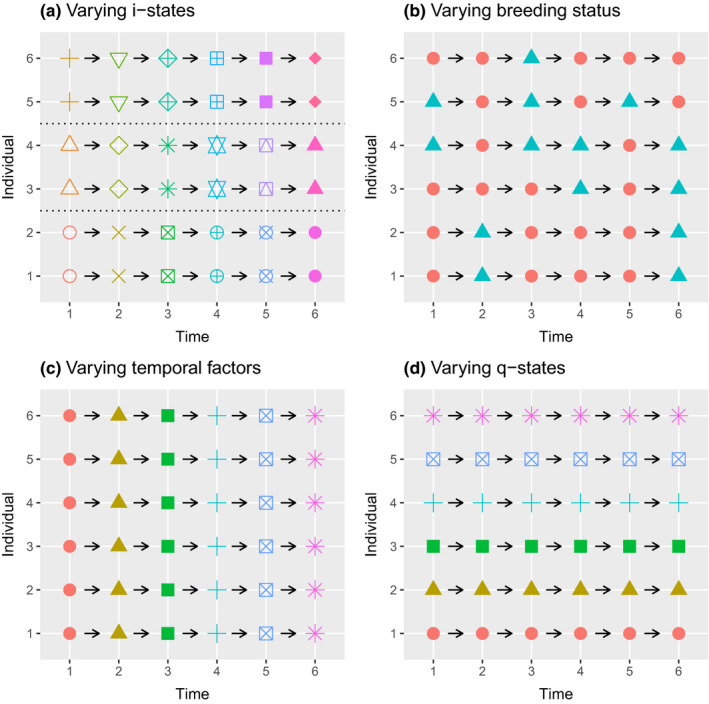
Growth rate, $g\left(\cdot \right)$, of individuals: (a) $g\left(\cdot \right)$ depends on the i‐states only, hence is constant within a group of individuals sharing the same i‐states (model I1); (b) $g\left(\cdot \right)$ depends on the breeding status only, hence is constant within the breeding group and the nonbreeding group (model D1a,D1b); (c) $g\left(\cdot \right)$ depends on the temporal factor only, hence is constant across individual but varying across time (model I2,D2a,D2b); (d) $g\left(\cdot \right)$ depends on the q‐states only, hence is varying across individual but constant across time (model I3,D3)

In Figure 1, we present a graphical representation of the differences between the proposed heterogeneity models. In each of the four scenarios, the individual growth model, $g\left(\cdot \right)$, depends on exactly one factor.

#### 2.3.5 Hybrid models

The proposed models can occur individually or be combined within and/or between the categories (labile individual, temporal, and persistent individual). For instance, combining models within the temporal category uses the correlated random year effects to explain the unaccounted correlation by the observed drivers. Alternatively, combining models between the labile individual and persistent individual heterogeneity accounts for two axes of correlations in one model. These different forms of combination of models expand the possibility of IPMs with nonindependent vital rates.

### 2.4 Numerical implementation

#### 2.4.1 Parameter estimation of vital rate models

In this paper, the vital rate models are fitted using the Markov chain Monte Carlo (MCMC) algorithms (Brooks et al., 2011) in NIMBLE (de Valpine et al., 2017, 2020a, 2020b) given individual‐level demographic data. Different from the usual approach in IPMs that each vital rate model is fitted separately, the proposed dependent models may require a joint estimation with multiple vital rate models. This may hence increase the computational cost and change the mixing behaviour of the MCMC algorithm.

Random effects in the models (I2,I3,D2b,I3) are treated as unobserved parameters, or auxiliary variables, and sampled within each iteration of the MCMC algorithm. Similarly, the auxiliary variables in the copula model (D2a) are sampled as unobserved parameters in the MCMC algorithm. We note that the random effects for the temporal and individual random effects induce very different mixing properties.

Prior distributions for all parameters are set to be noninformative and are presented in Appendix S2. We use the trace plot and Brooks‐Gelman‐Rubin statistic to assess convergence (Gelman & Shirley, 2011). Chains with a value of Brooks‐Gelman‐Rubin statistic being less than 1.05 are treated as converged.

#### 2.4.2 Approximation of $\log\lambda_{s}$

We use the asymptotic log population growth rate, logλ, as one metric to compare models. Mathematically, λ is defined as ${\text{lim}}_{t\to \infty }\left(N_{t+1}/N_{t}\right)$, where $N_{t}$ is the population abundance and can be approximated by solving the integral in Equation (2). It has been shown that logλ converges asymptotically, even in the temporally stochastic case (Ellner & Rees, 2007).

The log population growth rate of IPMs without temporal heterogeneity can be approximated via the midpoint rule (Easterling et al., 2000). To briefly illustrate the mid‐point rule, the projection kernel is discretized into a projection matrix by a sufficient number of mesh points that are of uniform length to discretize $\left(x,z\right)$ (Ellner & Rees, 2006). The population growth rate is then obtained as the leading eigenvalue of the projection matrix (Caswell, 2001). Alternatively, we can consider using mesh points that are uniform quantiles of z as the distribution of z is known.

However, when the IPMs include temporal heterogeneity, the midpoint rule becomes inapplicable. In this case, we use the simulation technique of “element‐selection” to approximate the log population growth rate (Ellner & Rees, 2007; Rees & Ellner, 2009). This approach creates a series of projection matrices, $K_{t}$ with the population abundance $N_{t}$ obtained by repeatedly multiplying the projection matrices with a discrete approximation of $n\left(x,z,t\right)$. The (stochastic) log population growth rate is approximated using the empirical mean given by,

(23) $$\begin{array}{c} \hat{\text{log}\lambda_{s}}\left(L,L_{0}\right)=\frac{1}{\left(L‐L_{0}\right)}\sum_{t=L_{0}}^{L‐1}\text{log}\left(\frac{N_{t+1}}{N_{t}}\right)=\frac{1}{\left(L‐L_{0}\right)}\text{log}\left(\frac{N_{L}}{N_{L_{0}}}\right), \end{array}$$

where data in the first $L_{0}<L$ years are excluded as transient dynamic to reduce the influence of random initialization. We note that this estimator carries an extra variability caused by finite simulation. Ellner and Rees (2007) showed that the estimator converges to a normal distribution such that,

(24) $$\begin{array}{c} \hat{\text{log}\lambda_{s}}\left(L,L_{0}\right):N\left[\text{log}\lambda_{s},\frac{1}{\left(L‐L_{0}\right)}\text{Var}\left\{\text{log}\left(\frac{N_{t+1}}{N_{t}}\right)\right\}{|}_{t=L_{0},\ldots ,L‐1}\right]. \end{array}$$

In addition to the $\text{log}\lambda_{s}$ itself, we are also interested in the variability on $\text{log}\lambda_{s}$ caused by parameter uncertainty. This parameter uncertainty can be easily propagated within the Bayesian framework since we are able to obtain samples from the posterior distribution of the parameters, which in turn can be used to calculate the value of logλ and hence obtain summary statistics of the posterior distribution.

#### 2.4.3 Sensitivity and elasticity analysis

We also estimate the sensitivity and elasticity of the asymptotic log growth rate, $\text{log}\lambda_{s}$, with respect to selected vital rate parameters (Rees & Ellner, 2009; Tuljapurkar, 1990; Vindenes et al., 2014). In particular, we note that Coulson et al. (2005) suggests that models incorporating between‐process correlations may alter the sensitivity estimate, which in turn has implication for management decisions. Here, we apply a central‐differencing approach to approximate the sensitivity such that,

(25) $$\begin{array}{c} \frac{\partial \lambda_{s}}{\partial \beta }=\frac{\lambda_{s}\left(\beta +\varepsilon \right)‐\lambda_{s}\left(\beta ‐\varepsilon \right)}{2\varepsilon }, \end{array}$$

where $\lambda_{s}\left(\beta +\varepsilon \right)$ is the estimate of $\lambda_{s}$ when the target parameter equals to β+ε. By running preliminary tests, we found that ε=0.005β is small enough to give precise estimate for all sensitivities of interest. Given the estimate of sensitivity, elasticity of β is obtained as,

(26) $$\begin{array}{c} \frac{\partial \lambda_{s}}{\partial \beta }\frac{\beta }{\lambda_{s}}. \end{array}$$

We note that the sensitivities/elasticities of the copula model (D1b) are the same as for the vanilla model (I1), similar to λ. To see this, we derive the analytical equations of sensitivity (see chapter 4 of Ellner et al., 2016) such that,

(27) $$\begin{array}{c} \frac{\partial \lambda_{s}}{\partial \beta }=\iint \frac{\partial \lambda_{s}}{\partial k(x^{\prime }|x)}\frac{\partial k(x^{\prime }|x)}{\partial \beta }dx^{\prime }dx, \end{array}$$

where both terms in the integral remain unchanged because the copula model does not distort the marginal vital rate models.

### 2.5 Simulation study

We conducted a simulation study to investigate how sensitive the summary statistics (logλ and elasticities) are to the different kinds of vital rate heterogeneity for parameters relevant to the Soay sheep example below. For target parameters of interest that toggle among models, we considered 2–3 values of interest, including a 0 value to compare with a simpler model. For example, model I2 (independent temporal heterogeneity) can be compared with model D2b (correlated temporal heterogeneity) by setting ρ to 0 (I2) or nonzero (D2b). Other parameters were either randomly generated from chosen distributions with 100 replications (Table 1) or fixed (Table 2). Randomly generated parameters allowed us to look at how summary statistics change over small ranges of variation in a coarse way, without looking at changes in relation to each parameter one by one. The distributions and values are motivated from the data in the case study, but slightly adjusted to show the difference between models with and without correlations.

**TABLE 1:** Random parameters

	Distributions
$\beta_{s,0}$	$N\left(‐4.25,0.05^{2}\right)$
$\beta_{s,m}$	$N\left(1.92,0.01^{2}\right)$
$\beta_{b,0}$	$N\left(‐1.47,0.05^{2}\right)$
$\beta_{b,m}$	$N\left(0.50,0.01^{2}\right)$
$\beta_{g,0}$	$N\left(1.20,0.05^{2}\right)$
$\beta_{g,m}$	$N\left(0.63,0.01^{2}\right)$
$\beta_{h,0}$	$N\left(0.46,0.05^{2}\right)$
$\beta_{h,m}$	$N\left(0.57,0.01^{2}\right)$

**TABLE 2:** Fixed parameters

	Values
$\beta_{g,q}$	0.01
$\sigma_{g}^{2}$	$0.09^{2}$
$\sigma_{h}^{2}$	$0.2^{2}$
$\nu_{g}^{2}$	$0.03^{2}$
$\nu_{b}^{2}$	$0.45^{2}$
$\theta_{g}^{2}$	$0.03^{2}$
$\theta_{b}^{2}$	$0.45^{2}$

The simulation study looks at theoretical behavior of the IPM models, not at statistical properties of parameter estimation. It reveals how model summary statistics shift with particular parameters, but not how parameter estimation performs if the wrong model is fitted to the data. Within the simulation study, we compare the independent models $\left(I1‐I3\right)$ and three of the dependent models $\left(D2a,D2b,D3\right)$. We do not include the models with labile individual heterogeneity as: (i) the impacts on logλ by the reproduction conditional models $\left(D1a\right)$ are always negative when $\beta^{\prime }<0$, and (ii) the copula model $\left(D1b\right)$ and vanilla model $\left(I1\right)$ are theoretically equivalent due to the unchanged marginal property (given the same parameter values). For models with temporal heterogeneity, we set $L_{0}=1000$ and L=10,000.

### 2.6 Soay sheep case study

We apply the different models to data on Soay sheep. The individual‐level demographic data consist of information from marked female sheep in the Village Bay area on the island of Hirta in the St. Kilda archipelago, Scotland, from 1986 to 1996. Details of the Soay sheep and data collection protocol can be found in Clutton‐Brock and Pemberton (2004), and the data are available from Coulson (2012).

Using preliminary runs for the estimation of parameters of the vital rate models, we set the burn‐in and total iteration numbers for the MCMC algorithm to be 20,000 and 100,000 for the majority of the models; for the random individual effects models, we used 40,000 and 200,000 (uncorrelated case, I3) and 200,000 and 1,000,000 (correlated case, D3). For the shared driver model $\left(D2a\right)$, we consider the winter North Atlantic Oscillation index (NAO) as the additional covariate (Clutton‐Brock & Pemberton, 2004). We follow Simmonds and Coulson (2015) and apply the average NAO for December, January, February, and March as the covariate, which are obtained from the Climate Research Unit at the University of East Anglia. For the distributions of NAO, we apply a normal distribution with mean ‐0.019 and standard deviation 1.09. For the copula model $\left(D1b\right)$, parameter α denotes the off‐diagonal element of the covariance matrix D in the multivariate Gaussian distribution. For the reproduction conditional model $\left(D1a\right)$, exploratory data analysis using a grid‐search approach suggested that newborns are likely to suffer from reduced growth in relation to reproduction. Thus, we refine the reproduction conditional model such that $\beta_{g|r}$ only accounts for the reduced growth of newborns in the growth model.

In addition, individual‐level demographic data of the case study contain missing data. For instance, we lack reproduction records of some marked individuals in the survey. This poses challenge on the proposed models that intend to capture the correlation between reproduction and growth. In this article, we analytically marginalise out the missing data to estimate parameters of interest.

## 3 RESULTS

### 3.1 Simulation study

**FIGURE 2:**
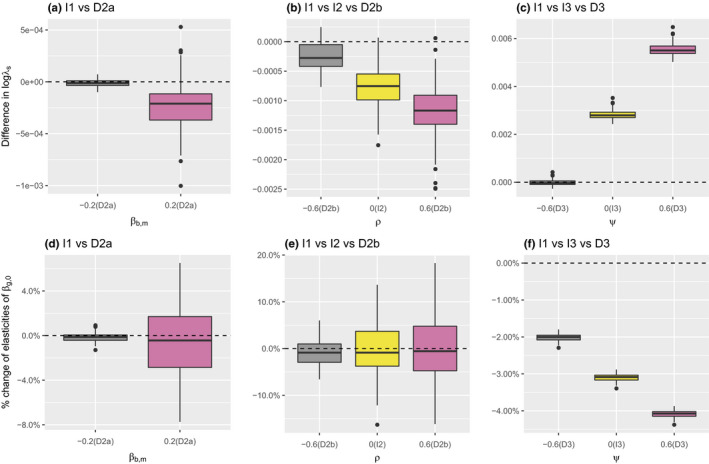
Comparison across models in simulation with 100 replications: (a) $\text{log}\lambda_{s}$(D2a) ‐ $\text{log}\lambda_{s}$(I1); (b) $\text{log}\lambda_{s}$(I2,D2b) ‐ $\text{log}\lambda_{s}$(I1); (c) $\text{log}\lambda_{s}$(I3,D3) ‐ $\text{log}\lambda_{s}$(I1); (d) % change of elasticity of $\beta_{g,0}$ of model D2a over model I1; (e) % change of elasticity of $\beta_{g,0}$ of model I2,D2b over model I1; and (f) % change of elasticity of $\beta_{g,0}$ of model I3,D3 over model I1. The dashed line is the reference line for I1

In Figure 2, we present the pairwise results of the vanilla model (I1) and the proposed (in)dependent models (I2,I3,D2a,D2b,D3). The models are compared with respect to $\text{log}\lambda_{s}$ (top row) and elasticities of growth intercept (bottom row) with known vital rate parameters.

Our simulations show that the variability of the given estimated quantities generally increases with increasing correlation in almost all scenarios; the exception is Figure 2(f) where the correlation appears to have little impact on the variability. The increase in variability is more substantial for models with temporal heterogeneity, especially the shared driver model $\left(D2a\right)$. Further, we observe that correlation in both forms of heterogeneity can lead to both increased or decreased values $\text{log}\lambda_{s}$ (Figure 2a–c). This is in line with the result that although uncorrelated temporal heterogeneity is generally predicted to decrease $\text{log}\lambda_{s}$, correlated temporal heterogeneity can increase $\text{log}\lambda_{s}$ (Doak et al., 2005; Fieberg & Ellner, 2001). Also, the temporal heterogeneity models and persistent individual heterogeneity model cause different impacts on $\text{log}\lambda_{s}$. For example, temporal heterogeneity appears to lead to reduced $\text{log}\lambda_{s}$; similarly, increasing the correlation in temporal heterogeneity models leads to a decrease in $\text{log}\lambda_{s}$ (Figure 2a,b). However, persistent individual heterogeneity models have the reverse effects (Figure 2c). Finally, we note that the trend on $\text{log}\lambda_{s}$ against correlation does not translate into that of elasticities. The decreasing trend of the temporal heterogeneity disappears (Figure 2a,b,d,e), while the trend of the persistent individual heterogeneity is reversed (Figure 2c,f).

### 3.2 Case study on Soay sheep

In Appendix S3, we present the posterior summary estimates of the model parameters for different models. Three dependent models (D1a,D2b,D3) indicate a significant correlation between growth and reproduction (the symmetric 95\% credible intervals of $\alpha ,\beta_{b,q}$ in model D1b,D2a contain 0). The reproduction conditional model (D1a) and the correlated random individual effects model (D3) indicate a negative association between growth and reproduction (${\hat{\beta }}_{g|r}<0,\hat{\psi }<0$), while the correlated random year effects model (D2b) estimates a positive correlation ($\hat{\rho }>0$). Note that these results in different sign of correlation do not contradict with each other because these models are driven by different biological mechanisms.

#### 3.2.1 Comparison of ${\log\lambda }_{s}$

We use 500 parameter values sampled from the posterior distribution to approximate the (stochastic) log population growth rate. The uncertainty from parameter estimation are hence propagated into the posterior distribution of $\text{log}\lambda_{s}$. In the temporally stochastic models, we set $L_{0}=1,000$ and L=10,000 to approximate $\text{log}\lambda_{s}$. Table 3 provides the corresponding summary statistics of $\text{log}\lambda_{s}$ for each model.

**TABLE 3:** Summary statistics of the (stochastic) log population growth rate with parameter uncertainty on Soay sheep

	Mean	95% credible interval
I1	0.0301	(0.0005, 0.0565)
I2	0.0380	(−0.0062, 0.0846)
I3	0.0312	(0.0022, 0.0562)
D1a	0.0330	(0.0048, 0.0598)
D1b	0.0394	(−0.0003, 0.0706)
D2a	0.0368	(0.0074, 0.0648)
D2b	0.0358	(−0.0054, 0.0790)
D3	0.0292	(0.0017, 0.0554)

We first observe that the mean of $\text{log}\lambda_{s}$ ranges approximately from 0.03 to 0.04, which translates into a 3 to 4% annual population growth rate. There is considerably more variability, however, in the uncertainty about $\text{log}\lambda_{s}$. In particular, the width of the credible intervals of $\text{log}\lambda_{s}$ by models with random year effects (I2, D2b) are around 35\% larger than that of the rest of the models. Second, we observe that the uncertainty on $\text{log}\lambda_{s}$ caused by parameter uncertainty is larger than the bias caused by ignoring the correlation structure. This is similar to the empirical result of Compagnoni et al. (2016) that parameter uncertainty outweighs the bias caused by ignoring the correlation structure. Further, we note that logλ of the vanilla model (I1) and the copula models (D1b) are slightly different despite the theoretical equivalence between the IPMs. This is because the parameter estimates between the models are different.

Finally, we note that the predictions of the shared drivers IPM (D2a) depend on the distribution of the winter NAO. Adjusting the distribution of the winter NAO may lead to different distributions of $\text{log}\lambda_{s}$ hence interpretation. In Appendix S4, we consider three other distributions obtained by using a nonparametric bootstrapping approach of the NAO in different years.

#### 3.2.2 Comparison of elasticity

We approximate the elasticities of four parameters, again using the sampled parameter values from the posterior distribution, presented in Table 4. We observe that models with random temporal effects lead to a larger variability in the elasticities, which is similar to the observation in $\text{log}\lambda_{s}$. Additionally, we note that the correlated random individual effects model $\left(D3\right)$ consistently gives different results across all four elasticities of interest. This leads to the interesting result that different models of nonindependence among demographic rates may yield different elasticities even when the $\text{log}\lambda_{s}$ is quite similar (Table 3).

**TABLE 4:** Summary statistics of elasticities of four selected parameters with parameter uncertainty on Soay sheep

	$\beta_{g,0}$	$\beta_{g,m}$	$\beta_{b,0}$	$\beta_{b,m}$
I1	1.6312	1.7602	−0.5519	0.5083
	(1.451, 1.787)	(1.516, 1.990)	(−0.675, −0.451)	(0.402, 0.630)
I2	1.5941	1.7253	−0.5213	0.4856
	(**1.384, 1.823**)	(**1.454, 1.989**)	(**−0.691, −0.359**)	(**0.300, 0.642**)
I3	1.5888	1.5793	−0.5506	0.5058
	(1.410, 1.752)	(1.325, 1.863)	(−0.673, −0.443)	(0.391, 0.632)
D1a	1.6381	1.7020	−0.5520	0.5097
	(1.463, 1.801)	(1.487, 1.916)	(−0.675, −0.458)	(0.413, 0.629)
D1b	1.6142	1.7561	−0.5527	0.5121
	(1.417, 1.774)	(1.504, 2.021)	(−0.658, −0.452)	(0.410, 0.608)
D2a	1.6606	1.7721	−0.5548	0.5175
	(1.479, 1.831)	(1.553, 2.008)	(−0.673, −0.455)	(0.417, 0.631)
D2b	1.6212	1.7725	−0.5424	0.5047
	(**1.376, 1.865**)	(**1.483, 2.067**)	(**−0.754, −0.322**)	(**0.290, 0.698**)
D3	*1.6878*	*1.6604*	*−0.6238*	*0.5819*
	(1.523, 1.856)	(1.436, 1.907)	(−0.757, −0.507)	(0.461, 0.714)

Note

Present are posterior mean and 95% credible interval. Note that models with random year effects (I2,D2b) usually have larger variability (in bold), and model D3 yields different elasticities (in italics).

## 4 DISCUSSION

### 4.1 Model summary

In this paper, we have presented a general framework and several specific approaches to modelling between‐process dependencies in IPMs. In particular, motivated by reproduction cost, we propose three categories of models (labile individual, temporal, and persistent individual heterogeneity) that reflect different biological mechanisms for the correlation structure between growth and reproduction.

Unlike independent IPMs, these modelling approaches explicitly characterise the dependency between vital rates, permitting the quantification of between‐process correlation. As a data‐driven method, this is better than assuming either no correlation, or perfect correlation across vital rates, i.e., assuming the correlation coefficient to be 1 or ‐1 (Benton & Grant, 1999; Coulson et al., 2011).

Amongst the proposed methods, application of the copula method for modelling vital rates is novel to IPMs. However, given the same estimates for the common parameters, the dependence structure of an IPM using copula models may lead to theoretically equivalent projections as the independent (vanilla) IPM. This is because (i) correlations in the copula model do not modify the marginal distributions and (ii) the involved vital rate models (reproduction and growth in our analysis) have an additive structure. In practice, however, copula IPMs will still differ from the vanilla IPMs due to differences in parameter estimates. Further, such theoretical equivalence will not remain with alternative copula structures, for example, when we consider the previous breeding status $\left(r_{j,t}\right)$ as opposed to the current breeding status $\left(r_{j,t+1}\right)$ in the copula structure with the growth vital rate. It may be appropriate to condition on reproduction at time t for some species, particularly when multiple reproduction‐related activities can cause energy loss in the parents including mating, gestation, parturition, lactation, etc (Gittleman & Thompson, 1988). Also, copula models can be applied to other aspects of IPMs. For instance, the multidimensional random effect distribution can be constructed by copula models, which bring extra flexibility to the models. The use of copula models within this general context is an area of current research.

### 4.2 Simulation and case study

In the case study of Soay sheep, the different IPM structures yielded relatively similar population estimates. This is most likely because the parameter uncertainty (which was ignored in the simulation studies) outweighed the impact of between‐process correlation (Compagnoni et al., 2016). In contrast, the results for both the simulation and the case study show that (i) different models for dependence between vital rates can yield similar (nearly identical) $\text{log}\lambda_{s}$, but different elasticities and (ii) variability of the population statistics are moderately affected by the correlation between vital rates.

Random effect models are commonly used to model dependence structures (Dingemanse & Dochtermann, 2013; Vindenes et al., 2014). Based on the simulation study, it appears that temporal and persistent heterogeneity can lead to differences in the estimated target statistics and their associated variability. Results suggest that the variability increases as the correlation increases. This aligns with the general understanding that extreme values are more likely to be generated, and hence, the variability of the target statistics increases when the correlation is large and positive (Doak et al., 2005; Fieberg & Ellner, 2001). Empirical results about the correlation in temporal variation have been discussed previously (Hindle et al., 2018; Metcalf et al., 2015). Additional random effects models can also be investigated, given available data, for example, allowing for nested spatial heterogeneity (Olsen et al., 2016), or independent/crossed structure of spatial and temporal heterogeneity (Jacquemyn et al., 2010). Such heterogeneity structures can provide additional flexibility and more complicated correlations in vital rates and hence IPMs.

### 4.3 Recommendation

In practice, model selection procedures are often carried out to determine whether one model is preferable to all others. However, we note that some of the proposed methods (D1a,D1b) do not allow unbalanced data, whereas other proposed methods (D2a,D2b,D3) are flexible for unbalanced/balanced data (Verbeke et al., 2014). Such differences complicate model selection, which usually assumes the competing models use the exactly same data. This is an area for future research.

In general, incorporating these five (biologically/statistically) distinct methods (in hybrid/separately) in IPMs may be beneficial. Although the correlations have little impacts on some statistics of interest (e.g., $\text{log}\lambda_{s}$), our empirical results show that elasticities of the unknown parameters and the associated variability are moderately affected by these correlations. These results may provide insights into the relationship between the possible dependencies on individual‐level vital rates and target population statistics. Therefore, we conclude that including such dependent structures is generally advisable when fitting IPMs to ascertain whether or not such between vital rate dependencies exist, which in turn can have subsequent impact on population management or life‐history evolution.

## CONFLICT OF INTEREST

The authors declare no conflict of interest.

## AUTHOR CONTRIBUTIONS

**Yik Leung Fung involved in** conceptualization (equal), formal analysis (equal), methodology (equal), visualization (equal), writing—original draft (equal), and writing—review and editing (equal). **Ken Newman involved in** conceptualization (equal), methodology (equal), supervision (equal), writing—original draft (equal), and writing—review and editing (equal). **Ruth King involved in** conceptualization (equal), methodology (equal), supervision (equal), writing—original draft (equal), and writing—review and editing (equal). **Perry de Valpine involved in** methodology (equal), validation (lead), visualization (equal), and writing—review and editing (lead).

## Supporting information

Appendix S1

Appendix S2

Appendix S3

Appendix S4

## Data Availability

The demographic data that support the findings of this study are openly available at http://www.oikosjournal.org/sites/oikosjournal.org/files/appendix/sheep_data_1986_to_1996.csv from Coulson, 2012. The NAO data that support the findings of this study are openly available at https://crudata.uea.ac.uk/cru/data/nao/nao.dat. The example code that support the findings of this study are openly available at https://github.com/EddieFung/IPM‐non‐independent‐vital‐rates.
