# Supplementary material for: Building integral projection models with nonindependent vital rates
Source: Ecol Evol. 2022 Mar 21;12(3):e8682. doi: 10.1002/ece3.8682 (PMC8935301; doi:10.1002/ece3.8682)
Supplement: Supplementary file 1 — Appendix S1 [file ECE3-12-e8682-s002.pdf]

S1 Derivation of the identical projection kernel with copula models

The projection kernel of the vanilla model ($I1$) is identical to the copula models ($D1b$). This can be seen as follows,

$$\begin{aligned} k(m' \mid m) &= \int s(m)\Big[b(m)\big\{h(m' \mid m) + f(m', x \mid r=1, m)\big\} + \big\{1 - b(m)\big\} f(m', x \mid r=0, m)\Big] dx \\ &= s(m) \int \Big[b(m)h(m' \mid m) + f(m', x, r=1 \mid m) + f(m', x, r=0 \mid m)\Big] dx \\ &= s(m)\Big[b(m)h(m' \mid m) + \int f(m', x \mid m) dx\Big] \\ &= s(m)\Big[b(m)h(m' \mid m) + f(m' \mid m)\Big] \\ &= s(m)\Big[b(m)h(m' \mid m) + g(m' \mid m)\Big], \end{aligned} \tag{S1.1}$$

where the last equality holds because the copula structure does not distort the marginal model of m', i.e., $f(m'|m) = g(m'|m)$. In contrast, for example, model $D1a$ changes the marginal of m' into a bimodal distribution hence the last equality does not hold.
