# Supplementary material for: Building integral projection models with nonindependent vital rates
Source: Ecol Evol. 2022 Mar 21;12(3):e8682. doi: 10.1002/ece3.8682 (PMC8935301; doi:10.1002/ece3.8682)
Supplement: Supplementary file 2 — Appendix S2 [file ECE3-12-e8682-s001.pdf]

S2 Prior distributions of parameters

We set the prior distributions for all parameters to be uninformative. Prior distribution on the inverse of the variance covariance matrix of the random effects is set to be Wishart distribution. For example, the prior distribution on random year effects model is

$$\begin{pmatrix} \nu_g^2 & \rho\nu_g\nu_b \\ \rho\nu_g\nu_b & \nu_b^2 \end{pmatrix}^{-1} \sim W\left(\begin{bmatrix} 0.001 & 0 \\ 0 & 0.001 \end{bmatrix}, df = 3\right),$$

where $W(\Omega, df)$ is the Wishart distribution with scale matrix Ω, degree of freedom df. For the remaining parameters, a single dimensional prior is given in Table S2.1.

	Prior Distribution
$\beta_{b,0}, \beta_{b,m}, \beta_{g,0}, \beta_{g,m}$<br>$\beta_{s,0}, \beta_{s,m}, \beta_{h,0}, \beta_{h,m}$<br>$\beta_{b,q}, \beta_{g,q}, \beta_{g\|r}$	$N(0, 100^2)$
σ_g^2, σ_h^2	$\Gamma^{-1}(0.001, 0.001)$

Table S2.1: Prior distributions for the remaining parameters
