# Supplementary material for: Building integral projection models with nonindependent vital rates
Source: Ecol Evol. 2022 Mar 21;12(3):e8682. doi: 10.1002/ece3.8682 (PMC8935301; doi:10.1002/ece3.8682)
Supplement: Supplementary file 3 — Appendix S3 [file ECE3-12-e8682-s003.pdf]

S3 Posterior summary of parameters for the fitted Soay sheep models

Posterior means and 95% symmetric credible intervals for parameters for the different models fit to the Soay sheep data are shown in Table S3.1. Table S3.2 provides the same information for the parameters related to survival and inheritances, which are the same across all models.

	Models							
	$I1$	$I2$	$I3$	$D1a$	$D1b$	$D2a$	$D2b$	$D3$
$\beta_{g,0}$	1.4935<br>[1.4155, 1.5697]	1.4959<br>[1.4235, 1.5699]	1.5589<br>[1.4684, 1.6458]	1.5174<br>[1.4371, 1.5981]	1.4965<br>[1.4206, 1.5734]	1.4963<br>[1.4175, 1.5733]	1.4853<br>[1.4085, 1.5682]	1.5657<br>[1.4799, 1.6544]
$\beta_{g,m}$	0.5298<br>[0.5044, 0.5559]	0.5305<br>[0.5068, 0.5543]	0.5073<br>[0.4780, 0.5378]	0.5225<br>[0.4957, 0.5491]	0.5325<br>[0.5061, 0.5565]	0.5277<br>[0.5020, 0.5540]	0.5334<br>[0.5066, 0.5584]	0.5061<br>[0.4762, 0.5348]
$\beta_{g,q}$						0.0055<br>[0.0009, 0.0101]		
$\beta_{g\|r}$				-0.0348<br>[-0.0667, -0.0027]				
σ_g	0.0882<br>[0.0836, 0.0932]	0.0862<br>[0.0816, 0.0912]	0.0814<br>[0.0755, 0.0879]	0.0885<br>[0.0835, 0.0932]	0.0890<br>[0.0841, 0.0946]	0.0878<br>[0.0833, 0.0928]	0.0862<br>[0.0815, 0.0910]	0.0811<br>[0.0754, 0.0869]
ν_g		0.0285<br>[0.0123, 0.0547]					0.0278<br>[0.0151, 0.0480]	
θ_g			0.0346<br>[0.0185, 0.0479]					0.0357<br>[0.0238, 0.0476]
$\beta_{b,0}$	-7.4546<br>[-8.763, -6.001]	-7.3736<br>[-8.924, -5.731]	-8.3763<br>[-10.215, -6.645]	-7.4620<br>[-8.739, -6.068]	-7.5832<br>[-8.865, -6.114]	-7.3742<br>[-8.841, -5.986]	-7.5424<br>[-8.888, -6.333]	-8.8491<br>[-10.766, -7.074]
$\beta_{b,m}$	2.2081<br>[1.7278, 2.6389]	2.1899<br>[1.6460, 2.6780]	2.4704<br>[1.9005, 3.0730]	2.2107<br>[1.7503, 2.6290]	2.2521<br>[1.7644, 2.6744]	2.2221<br>[1.7589, 2.7047]	2.2507<br>[1.7997, 2.7329]	2.6481<br>[2.0686, 3.2738]
$\beta_{b,q}$						-0.1385<br>[-0.219, 0.057]		
ν_b		0.5289<br>[0.2799, 0.9625]					0.3975<br>[0.1863, 0.6949]	
θ_b			0.7201<br>[0.4169, 0.9891]					0.6013<br>[0.2848, 0.8992]
α					-0.1652<br>[-0.389, 0.1919]			
ρ							0.6937<br>[0.0731, 0.9502]	
ψ								-0.8297<br>[-0.965, -0.505]

Table S3.1: Mean estimate and 95% credible interval of parameters in different models.

$\beta_{s,0}$	$\beta_{s,m}$	$\beta_{h,0}$	$\beta_{h,m}$	σ_h
-7.1113	2.8931	1.0124	0.4902	0.2270
[-8.3652, -5.9479]	[2.4812, 3.3335]	[0.5551, 1.4703]	[0.3419, 0.6386]	[0.2073, 0.2490]

Table S3.2: Mean estimate and 95% credible interval of parameters in survival and inheritance functions. α denotes the off-diagonal element of the covariance matrix D in the multivariate Gaussian distribution in the copula model ($D1b$).
