# Supplementary material for: Building integral projection models with nonindependent vital rates
Source: Ecol Evol. 2022 Mar 21;12(3):e8682. doi: 10.1002/ece3.8682 (PMC8935301; doi:10.1002/ece3.8682)
Supplement: Supplementary file 4 — Appendix S4 [file ECE3-12-e8682-s004.pdf]

S4 Growth rate of shared drivers models with various distributions of winter NAO

To investigate the impact on different distributions, Table S4.1 considers four distributions of the winter NAO, obtained by a normal distribution (Simmonds and Coulson, 2015), and using a non-parametric bootstrapping approach of the NAO in the survey years (1986-1996), the last 30 years (1990-2019), and 50 years (1970-2019).

	Mean	95% Credible Interval
N	0.0368	(0.0074, 0.0648)
SY	0.0317	(0.0014, 0.0575)
30	0.0329	(0.0031, 0.0587)
50	0.0334	(0.0040, 0.0592)

Table S4.1: Summary statistics of the (stochastic) log population growth rate with parameter uncertainty of shared drivers models on Soay sheep. The distributions are N: normal distribution; SY: bootstrapping the survey years; 30: bootstrapping 30 years; 50: bootstrapping 50 years
